# Time changes: Timing contexts support event segmentation in associative memory

**DOI:** 10.3758/s13423-021-02000-0

**Published:** 2021-10-13

**Authors:** Vincent van de Ven, Moritz Jäckels, Peter De Weerd

**Affiliations:** Department of Cognitive Neuroscience, Faculty of Psychology and Neuroscience, Maastricht University, P.O. Box 616, 6200 MD Maastricht, The Netherlands

**Keywords:** Timing context, Event segmentation, Subjective duration, Temporal memory, Associative memory

## Abstract

We tend to mentally segment a series of events according to perceptual contextual changes, such that items from a shared context are more strongly associated in memory than items from different contexts. It is also known that timing context provides a scaffold to structure experiences in memory, but its role in event segmentation has not been investigated. We adapted a previous paradigm, which was used to investigate event segmentation using visual contexts, to study the effects of changes in timing contexts on event segmentation in associative memory. In two experiments, we presented lists of 36 items in which the interstimulus intervals (ISIs) changed after a series of six items ranging between 0.5 and 4 s in 0.5 s steps. After each list, participants judged which one of two test items were shown first (temporal order judgment) for items that were either drawn from the same context (within an ISI) or from consecutive contexts (across ISIs). Further, participants judged from memory whether the ISI associated to an item lasted longer than a standard interval (2.25 s) that was not previously shown (temporal source memory). Experiment 2 further included a time-item encoding task. Results revealed an effect of timing context changes in temporal order judgments, with faster responses (Experiment 1) or higher accuracy (Experiment 2) when items were drawn from the same context, as opposed to items drawn from across contexts. Further, in both experiments, we found that participants were well able to provide temporal source memory judgments based on recalled durations. Finally, replicated across experiments, we found subjective duration bias, as estimated by psychometric curve fitting parameters of the recalled durations, correlated negatively with within-context temporal order judgments. These findings show that changes in timing context support event segmentation in associative memory.

It has been proposed that changes in a perceptual context of an ongoing experience can provide boundaries by which that experience can be segmented in contextually coherent chunks of events in memory (DuBrow & Davachi, 2016; Kurby & Zacks, 2008; Zacks et al., 2006). Such segmented chunks of events may serve as predictive models that can guide perception, action, and novel memory formation during future experiences. Behavioral and neuroimaging studies of event segmentation have shown enhanced memory for items with a shared visual or semantic context compared with items that come from different contexts (Ben-Yakov & Henson, 2018; DuBrow & Davachi, 2016; Heusser et al., 2018; Newtson & Engquist, 1976; Schwan & Garsoffky, 2004; Schwan et al., 2000; Speer et al., 2007; van Helvoort et al., 2020). The role of event segmentation in associative memory was aptly shown in a previous study (Heusser et al., 2018), in which participants saw multiple series of visual items while the context of the items (the color of a frame around the item) changed with each series. To test associative memory, participants judged the temporal order of items drawn from the previous series. Results showed better temporal order memory judgments for items that were selected from the same context, compared with order judgments for items that were selected from different contexts (i.e., crossing a change in contextual feature). In addition, participants also showed better memory for item–color associations when the item was the first of a new series (i.e., directly after a contextual change), compared with when it was presented later in the series. This finding suggests that the contextual change resulted in a transient increase of attentional capture of the visual environment, which enhances associative processing of items encountered during or right after the moment of the contextual change (Kentros et al., 2004; Lin et al., 2010). Thus, a change in context may lead to a trade-off of enhanced item-context associative processing against impaired processing of temporal order for items crossing those contextual changes.

Similar effects of segmentation have also been observed for changes in *temporal* contexts. Participants better encoded filmic or textual events at or shortly after a temporal contextual shift, compared with moments without a temporal shift (Magliano et al., 2001; Speer & Zacks, 2005), but also showed impaired temporal order memory for items crossing a temporal context change, compared with items with a shared temporal context (Ezzyat & Davachi, 2011). These findings are in line with the long held notion that time is an important organizing feature in associative memory (Eichenbaum, 2013; James, 1950; Tulving, 1984).

However, these event segmentation studies used symbolic cues to *imply* changes in temporal context, such as particular textual phrases in written stories (e.g., “A moment later”) or filmic features (e.g., change from day to night). Thus, segmentation based on symbolic temporal cues would be similar to segmentation based on nontemporal symbolic visual or auditory contextual cues. It remains unknown if and how the actual timing contexts of events, such as rhythm or duration, affect event segmentation (Rhodes, 2018). One notable difference between perceptual and timing contexts is that changes in perceptual cues are typically observed as instantaneous, whereas changes in timing context take time to be observed. Temporal event boundaries could thus be blurred or smeared across time, compared with changes in visual context, resulting in weaker or less detectable boundaries and subsequently less segmentation and reorganization of events in memory.

Further, it is well known that humans make systematic errors in their judgments of subjective duration, with duration estimations becoming more variable or imprecise with progressively longer time intervals (Buhusi & Meck, 2005; Gibbon et al., 1984; Rakitin et al., 1998; van Rijn et al., 2014). This asymmetric distribution of duration estimation error could impair mnemonic processing for events with longer timing contexts differently than those with shorter durations (Howard & Eichenbaum, 2013). More particularly, participants vary in their tendency to underestimate the actual length of a temporal duration (i.e., subjective duration estimation bias). This can be observed in duration discrimination tasks, in which participants must judge whether a time interval lasts longer than a standard interval (Rhodes, 2018; van Rijn, 2016). Participants with a higher underestimation bias are more likely to erroneously report a longer interval lasting shorter than the standard interval, especially when the difference between the two intervals is relatively small. Underestimation of temporal duration can be explained as a “mental” clock ticking at a slower pace, leading to diminished temporal discriminability (Gibbon et al., 1984; Killeen, 2002; van Rijn et al., 2014). This would make it more difficult to detect changes in timing contexts, resulting in weaker or more variable temporal boundaries for segmentation. In turn, a slower clock pace during encoding of temporally separated events could result in remembering those events as if they happened closer in time. If so, a stronger bias towards temporal underestimation would impair temporal memory for events that occurred with shorter intervals more than it would for events with longer intervals. In sum, the degree to which (changes in) timing contexts enhance associative processing within a shared context may depend on the accuracy of the mental representations of duration.

To study whether timing context plays a role in event segmentation, we adapted a previous study design (DuBrow & Davachi, 2013; Heusser et al., 2018) such that temporal regularity was the contextual feature. Participants saw multiple series of six items, with the interstimulus interval (ISI) remaining the same within a series but changing between series. Afterwards, we tested participants’ memory of the temporal order of a pair of items that was either taken from within the same or across different timing contexts. A difference in temporal order judgments between the two types of trials would be evidence that timing context supports event segmentation in a similar way as visual context. In a second memory test, we tested participants’ memory of the temporal source (i.e., ISI) of an item. Here, participants had to judge whether the ISI associated to a previously encoded item was longer in duration than a standard (fixed) duration of 2.25 s. This task follows the binary decision procedure of subjective duration estimation tasks, in which participants judge whether an interval of variable duration is longer than a standard (fixed) duration (Dyjas et al., 2012; Johnston et al., 2006; Matthews & Meck, 2016). In case of accurate temporal source memory, the proportion of “longer than standard” judgments should increase with the memory of increasing ISI. Further, the profile of judgments as a function of ISI can be well captured by fitting a cumulative Weibull distribution function, with “shape” and “slope” parameters of the function estimated as free parameters (Wichmann & Hill, 2001). The shape parameter can be regarded as the position of the midpoint of the curve on the *x*-axis, with increasing values indicating a higher tendency to underestimate temporal duration. The slope parameter indicates the steepness of the curve midpoint, which is related to the degree of subjective temporal discriminability of the memorized ISIs against the standard interval. To investigate whether underestimation or discriminability were associated to temporal order judgments, we correlated the parameters with accuracy of temporal order judgments for item pairs drawn from the same or different timing contexts.

The results of our experiment showed that changes in timing context could play a role in event segmentation. However, overall performance on the temporal order task was relatively low while the pattern of better within-context rather than across-context temporal memory was reflected in response times rather than accuracy. These unexpected findings limited interpretation of timing contexts in event segmentation. To address these issues and replicate our findings, we conducted a second experiment using a similar design but with slight modifications to enhance encoding and temporal memory performance. Results of the second experiment revealed better overall temporal memory performance while replicating the main findings of the first experiment, and allowed analysis of temporal boundaries during encoding.

## Experiment 1: Methods and materials

### Participants

Thirty-one participants (18 females, mean age = 22.1 years, *SD* = 2.8 years) were recruited via online social media and a student database at the Faculty of Psychology and Neuroscience. Participants who were diagnosed with a neurological or mental disorder, not well rested, under the influence of drugs or medication, or not between 18 and 45 years old were excluded. As compensation, particiants received a gift voucher worth €7.50 per hour, or course credit. The study was approved by the ethics review board of the Faculty of Psychology and Neuroscience. All participants gave written informed consent prior to the start of the experiment.

### Materials

A stimulus set of 750 unique visual images depicting different objects was selected from a publicly available data set (Kovalenko et al., 2012). For each participant, 12 lists of images of the full set were generated by random draw. Each of those lists included six series that each comprised six images, with no image being used twice for a participant. Thus, each participant saw 648 unique images. The experiment was coded in PsychoPy2 Version 1.84 (Peirce, 2007). Temporal accuracy of ISI and stimulus presentation was controlled using the frame-based presentation functionality of PsychoPy (Garaizar & Vadillo, 2014; Peirce, 2007). The testing was done on a PC running Windows 7 and a computer monitor with a 60-Hz refresh rate.

### Procedure

Participants first were instructed to encode and memorize as many of the presented images as they could. They then completed a short practice version of the task before the start of the experiment, in which they were exposed to one list of 36 items, and two subsequent memory tests (see below). Afterwards, participants were asked to verbally repeat the task instructions before proceeding with the main experiment.

In the main experiment, participants were presented with 12 lists of six series each, with each series comprising six images. A series was defined by a fixed ISI between the images within the series, such that a new series started with the presentation of a different ISI. ISIs for a series were sampled from a set of eight different time intervals that ranged from 500 to 4,000 ms, in steps of 500 ms. ISIs were pseudorandomized across series with the constraint that consecutive ISIs had to be at least 1s apart in duration, in order to increase the likelihood that participants could observe the changes in duration. During the ISI, a fixation cross was shown. Each item in a series was presented for 2,250 ms.

**Fig. 1:**
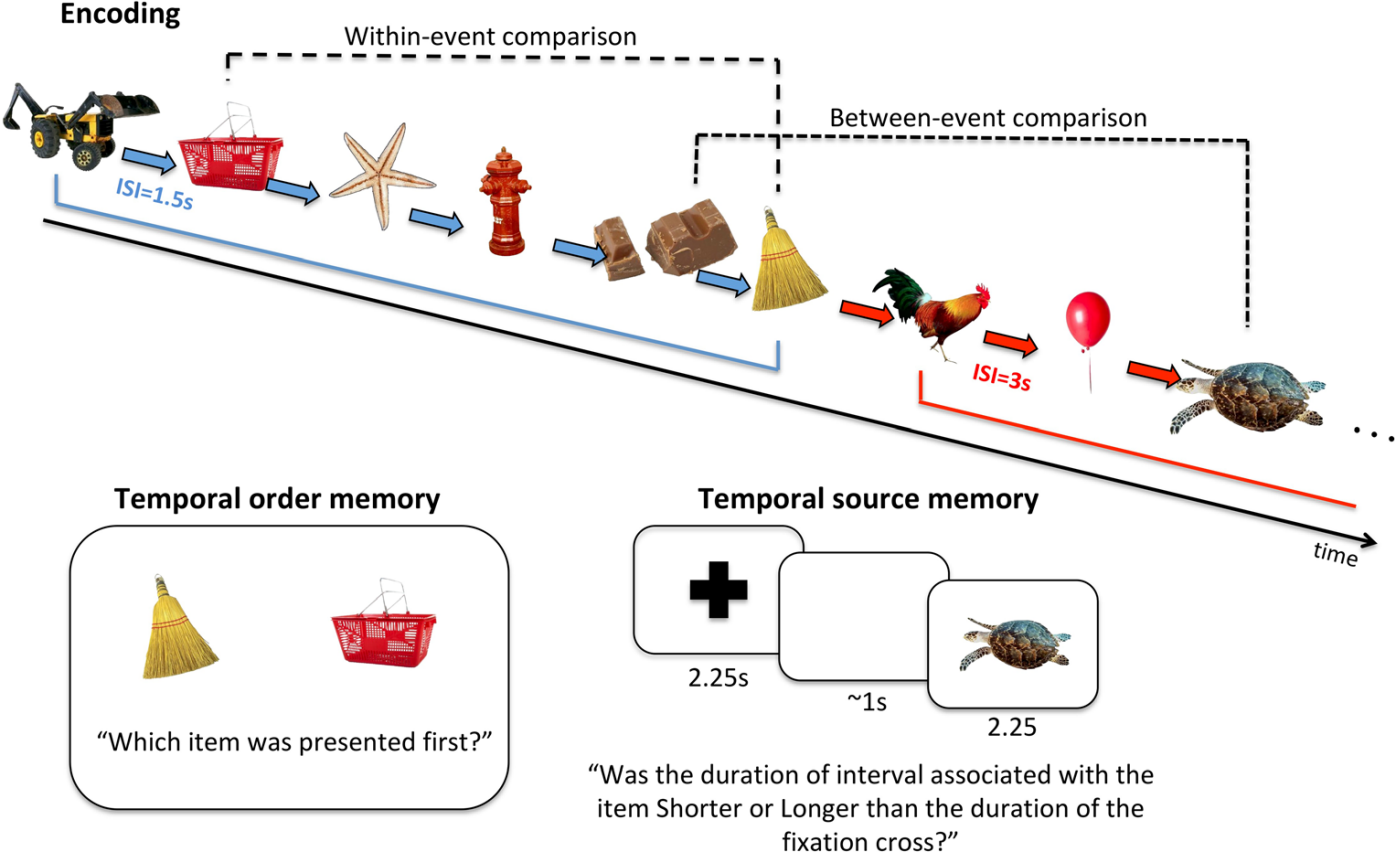
Experimental design. Participants encoded series of images in which interstimulus interval (ISI) changed every six items. Afterwards, participants completed a temporal order memory task and a temporal source memory task (see main text for details)

After viewing each list, participants completed two different memory tests. In trials of the temporal order memory Test (see Fig. 1), two images from the previous list were presented simultaneously at the left and right of the fixation cross, and participants had to judge which of the two appeared earlier within the list. Participants indicated their choice by pressing a button on the computer keyboard (“K” when they judged that the left image appeared first, or “L” otherwise). Every item pair was presented for 2.5 s, and assignment of location of the items to either the left or right of the fixation cross was randomized across trials. The items were selected from the list according to one of two conditions. In the *within-context* condition, an item from position two within a timing context was compared with an item of position six of the same context, whereas in the *across-context* condition, item five of one context was compared with item three of the subsequent context. Thus, the serial distance between two items in the temporal order memory test was the same for both conditions. Participants saw 10 temporal order trials after each list of images, with five pairs tested for each of the two conditions, resulting in a total of 60 trials per temporal order condition. To reduce recency effects, the items of the first half of the list were tested first, while the items of the second half were tested last (Heusser et al., 2018).

In the second test (temporal source memory task, TSMT; see Fig. 1), participants were tested on their memory of the duration of the ISIs associated with individual test items. On each trial, participants were first presented with a fixation cross for 2,250 ms (average duration of all ISIs), followed by an empty screen of 1,000 ms and then an item (for 2,250 ms) that was presented in the previous list. Participants were then asked to indicate whether the duration of the fixation cross was shorter or longer than the ISI preceding the image when it was presented in the just-shown list. To test the item–ISI association, items were pseudorandomly selected from each of the six positions with a uniform probability across all lists (each participant completed 216 temporal source memory trials in total across all positions and ISIs). Proportion of “longer than standard” responses (referred to as “longer” responses in remainder of the text) was then plotted as a function of ISI in order to obtain a psychometric response curve that described subjective duration based on temporal associative memory.

#### Analysis

Temporal order memory performance was analyzed with proportion correct answers and response times as dependent variables, using paired-samples *t* tests (two-tailed). Temporal source memory performance was analyzed with proportion of “longer” responses as dependent variable, using a repeated-measures analysis of variance (ANOVA) and paired-samples *t* tests. In addition, we analyzed temporal source memory performance by fitting the response proportions to a two-parameter cumulative Weibull function of the form:

1 $$F\left(x;\alpha, \beta \right)=1-{e}^{-{\left(\frac{x}{\alpha}\right)}^{\beta }},$$

with *x* denoting the proportion of “longer” responses for each ISI, and α and β, respectively, indicating the “shape” and “slope” parameters of the function. Parameter values were subsequently log-normalized to approximate a normal distribution. To assess whether memory for temporal order correlated with associative memory of temporal duration, we correlated the log-normalized parameter values with the temporal order accuracy scores. Curve fitting was done in MATLAB 2014a using a modified version of the *fminsearch* optimization routine. Statistical analysis was conducted using JASP (https://jasp-stats.org), and effects were deemed significant at an alpha of 0.05.

### Results

#### Temporal order memory

**Fig. 2:**
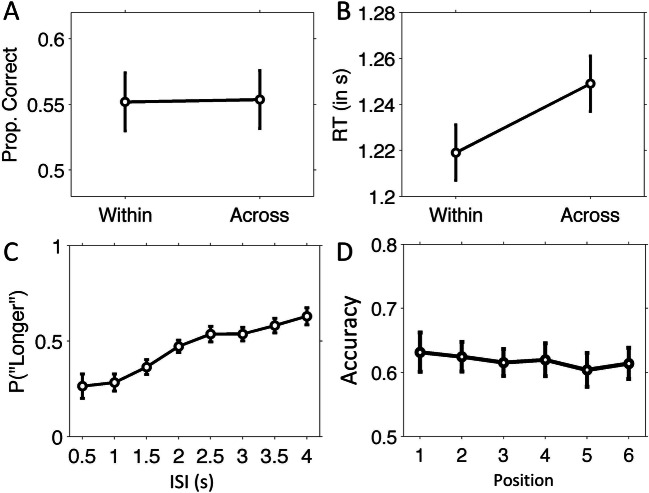
Temporal memory results of Experiment 1. Proportion correct (**a**) and response times in seconds (**b**) for the temporal order memory task. **c** Proportion “longer” responses during the temporal source memory task as a function of timing context during encoding. **d** Temporal source accuracy is plotted as a function of serial position. Error bars indicate 95% confidence intervals (Masson & Loftus, 2003)

Figure 2a shows the mean proportions of correct answers for both temporal order conditions. The performance of the Within-context condition (*M* = 0.55, *SD* = 0.10) was relatively low but significantly above chance (one-sample *t* test against 0.5), *t*(30) = 3.04, *p* = .005, as was performance of the across-context condition (*M* = 0.55, *SD* = 0.09), *t*(30) = 3.23, *p* = .003. Performance of the Within-context condition did not significantly differ from that of the across-context condition, t(30) = −0.08, *p* = .93. To verify that this null finding was not driven by the generally low performance, we repeated the analysis for a subset of the participants with above-chance accuracy (>0.5). In this subset (*N* = 22), performance of the within-context condition (*M* = 0.59, *SD* = 0.07), again did not differ, *t*(21) = 1.08, *p* = .29, from the across-context condition (*M* = 0.56, *SD* = 0.09).

For response times, we found significantly faster responses for correct temporal order judgments, *t*(30) = −4.31, *p* < .001, Cohen’s *d* = −0.31 (see Fig. 2b) in the within-context condition (*M* = 1.17 s, *SD* = 0.23), compared with the across-context condition (*M* = 1.23s, *SD* = 0.22). The effect size was comparable for the subset of participants with better overall performance, *t*(21) = −2.37, *p* = .028, Cohen’s *d* = −0.50, indicating that the effect was likely not due to guessing.

A possible concern could be that the limited response window (response time deadline) induced a speed–accuracy trade-off that affected the two conditions differently, thereby resulting in a similar pattern of response times but that was unrelated to temporal boundaries. To investigate this issue, we calculated the conditional accuracy function (CAF) as metric for speed–accuracy trade-off (Heitz & Engle, 2007). For each participant and condition, we rank-ordered and binned RTs in five equally sized quantiles, calculated the average accuracy for each RT quantile and fitted a second-order polynomial curve through the five estimated datapoints. A paired-sample *t* test of the curve fits between the two conditions was not significant, *t* (30) = −1.0, *p* = .32, indicating that speed–accuracy trade-off was unlikely to underlie the observed response times effect.

#### Temporal source memory

Figure 2c depicts the proportion “longer” responses that participants made on average for items from each of the eight ISIs. The proportions “longer” responses were lowest for items shown during the shortest interval of 0.5 s, and progressively increased for longer intervals. A one-way repeated-measures ANOVA showed that this effect was significant (Greenhouse–Geisser corrected for violation of sphericity), *F*(2.9, 86.9) = 37.29, *p* < .001, ${\eta}_p^2$ = 0.55, as was its linear contrast (*p* < .001). We then compared the “longer” proportions to the theoretical proportion of 0.5, which signifies indiscrimination about the comparison to the standard interval. Proportions were significantly below 0.5 (one-sample *t* tests) for intervals of 0.5, 1, and 1.5 s, and significantly higher for intervals 3.5 and 4 s (see Table 1). Hence, participants had more difficulty comparing the recalled interval durations closer to the standard duration of 2.25 s.

**Table 1:** Temporal source memory results Experiment 1

ISIs	Mean	*SE*	*t*	*p*	Cohen's *d*
0.5	0.26	0.03	−8.22	<.001	−1.48
1.0	0.28	0.02	−10.38	<.001	−1.86
1.5	0.36	0.03	−4.89	<.001	−0.88
2.0	0.47	0.03	−0.91	.370	−0.16
2.5	0.54	0.04	1.05	.304	0.19
3.0	0.54	0.03	1.23	.227	0.22
3.5	0.58	0.03	2.52	.017	0.45
4.0	0.63	0.03	4.01	<.001	0.72

*Note.* Means, *SEM*s and *t*-test results of the proportion “longer” responses against the hypothetical baseline of 0.5 (indecisiveness). Interstimulus intervals (ISIs) are listed in seconds.

We then analyzed whether the association between items and ISIs differed across item position within a list. To this end, we calculated accuracy for each of the six positions, pooled across ISIs, and analyzed these values using a one-factor repeated-measures ANOVA with 6 levels (Position 1 through 6; see Fig. 2d). We found no significant effect of item position, *F*(5, 150) = 0.22, *p* = 0.95, indicating that memory for ISI did not differ across the six serial positions in a list.

#### Correlation between psychometric curves and temporal order accuracy

In this analysis, we investigated whether performance on the two temporal memory tasks were related. While the difference between accuracy of within-context and across-context temporal order judgments was not significant, it is possible that some participants would show a “within-context benefit” if they were better able than others to perceive the changes in timing context. If so, then this variation could be related to individual differences in mental time keeping, in which a slower mental clock would make it more difficult to perceive changes in duration and thereby impede on temporal boundary processing and segmentation. We approximated individual differences in mental clock speed by psychometric curve fitting of the distribution of “longer” proportions for each participant.

**Fig. 3:**
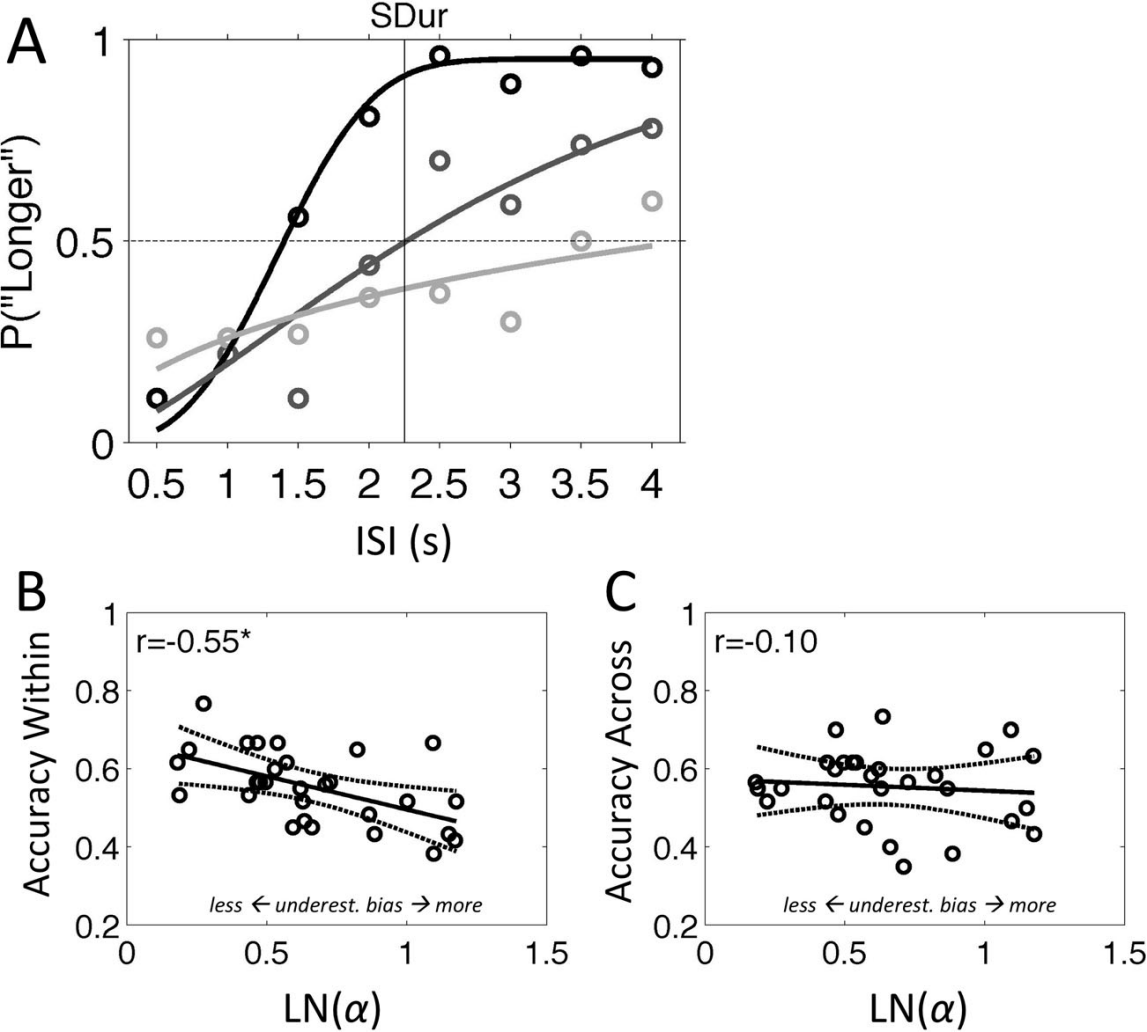
Temporal memory task correlations Experiment 1. **a** Psychometric curve (Weibull) fits to three participants (black, dark grey, and light grey). Dots indicate averages of observed datapoints. Correlation plots of the within-context (**b**) and across-context (**c**) temporal order judgments as a function of the log-normalized shape parameter, LN(*α*), of the Weibull curve fits. Higher LN(*α*) values indicate more bias towards temporal underestimation (underest. bias). Lines in the plots show the linear regression (straight line) and upper and lower 95% confidence intervals (dotted lines). ISI, interstimulus interval (in seconds); SDur, standard duration of 2.25 s; *p < .01; for the three participants shown in Panel **a**, LN(*α*) respectively is 0.19, 0.47, and 0.88, and LN(*β*) is 0.48, 0.15 and −0.24

The curve fitting procedure failed for two participants, leaving *N* = 29 for further analysis. Figure 3a shows the fitted psychometric curves for three participants with different response distributions. For the log-normalized shape parameter, ln*(α)*, the average (*SD*) was 0.66 (0.30), with a range of 0.18–1.18. The accuracy of Within-context temporal order judgments was significantly negatively correlated with ln*(α)* (*r* = −.55, *p* = .0022; see Fig. 3b), with participants showing lower accuracy also showing increased bias towards underestimation. For across-context judgments, accuracy did not correlate with ln*(α)* (*r* = −.10, *p* = .62; see Fig. 3d). Using a method to compare paired correlations (Meng et al., 1992), we found that the two correlations were significantly different (z = −2.04, *p* = .031, two-tailed; calculated in MATLAB using Spaak, 2012).

For the log-normalized slope parameter, ln(*β*), the average (*SD*) was −0.15 (0.37), with a range of −1.60–0.48. The slope parameter positively correlated with the within-context judgments (*r* = 0.35, *p* = .06) but not with the across-context judgments (*r* = .01, *p* = .94), and the two correlations did not significantly differ (*z* = 1.44, *p* = .13, two-tailed).

To verify that results were not driven by poor temporal order performance, we recalculated the correlations for the subsample of participants with above-chance temporal order performance. All previously significant correlations (including the difference between paired correlations) were also found to be significant for the subsample (within-context: *r* = −.48, *p* = .020; across-context: *r* = .24, *p* = .28; difference: *z* = −2.27, *p* = .017). In all, these findings thus showed that underestimation bias was inversely associated with temporal order accuracy for within-context items, but not for across-context items.

Furthermore, a slower mental clock underlying a stronger underestimation bias could result in items being perceived or remembered as having been presented closer in time. If so, then temporal order judgments for shorter ISIs may be less accurate than those for longer ISIs. To test this possibility, we compared accuracy for within-context temporal order judgments of short ISIs to that of long ISIs. To enhance the difference between short and long ISIs, we excluded the ISIs of 2.0 and 2.5 in this analysis. Results showed that within-context temporal order accuracy for short ISIs was lower (*M* = 0.57, *SD* = 0.10) than that for longer ISIs (*M* = 0.62, *SD* = 0.14), and this difference was statistically significant, *t*(30) = −1.83, *p* = .039, Cohen’s *d* = −0.33, one-tailed). This result was also replicated for the subsample of participants with above-chance performance, *t*(21) = 2.38, *p* = .027, and thus further supports the suggestion that changes in timing context facilitates event segmentation in memory formation.

We also analyzed the correlations of temporal order RTs with mnemonic duration performance measures. Correlations between ln(*α*) and RTs of the two temporal order conditions were not significant (all *p*s > .21), and neither were correlations between ln(*β*) and RTs (all *p*s > .75). We also correlated the curve fitting parameters with the response time difference between the two conditions (i.e., within–across) and found no significant correlations, ln(*α*): *r* = −.26, *p* = .18; ln(*β*): *r* = .25, *p* = .18. Finally, mean temporal source memory accuracy did not significantly correlate with the response time difference (*r* = .1, *p* = .62). These null results indicate that mnemonic duration judgments were not related to response times of the order judgments.

### Discussion

Our findings showed that changes in timing context affected mnemonic processing. We observed faster temporal order responses when items during testing were drawn from within the same timing context, compared with when crossing temporal boundaries. Notably, previous studies reported lower accuracy in across-context temporal order judgments, rather than response times (DuBrow & Davachi, 2013; Heusser et al., 2018). Nonetheless, longer response times as well as lower accuracy indicate worse temporal order memory for items crossing a contextual boundary, and suggest that changes in timing context affected processing of temporal order for items that crossed the temporal boundaries. We discuss this issue in more detail in the General Discussion.

Second, participants were on average well able to recall and compare the timing context of individual items to a standard duration stimulus, which indicated that they had acquired temporal source memory of the items during list viewing.

Third, we found that within-context, but not across-context, temporal order judgments were negatively correlated with the shape parameter of the psychometric curve, indicating that memory for items with a shared timing context may be related to an individual’s mental clock speed. This novel finding provides further evidence that changes in timing context affect associative memory processing, with an individual’s time estimation bias modulating how well temporal boundaries can be observed and thereby facilitate the within-context associative memory benefit. The marginally significant positive correlation between the slope parameter and within-context temporal order judgments further contributes to this suggestion, as it indicates better temporal order judgments when temporal discriminability tends to be higher.

However, interpretation of our findings is hampered by the relatively low accuracy of temporal order judgments. A previous study using a similar paradigm found average temporal order accuracies above .6 (Heusser et al., 2018). One important difference between this study and ours is their use of an explicit encoding task, which has been shown to enhance memory encoding of temporal order (DuBrow & Davachi, 2013; Sheldon, 2020). While we did not use an encoding task, participants were made aware of the post-encoding memory requirements through the experiment instructions and by completing a practice round that included the memory tasks. Nevertheless, the absence of a boundary-related effect on temporal order accuracy obfuscates interpretation of our results in terms of event segmentation.

To address these issues, we conducted a second experiment (Experiment 2) to replicate, substantiate and extend our current findings. In Experiment 2, we included a time–item associative encoding task in which participants rated how much they thought that duration was conceptually related (“appropriate”) to the visual item that followed that duration. Analysis of the encoding response times would allow us to test whether the first item of a timing context (i.e., temporal boundary item) was processed differently than other items in that context (temporal nonboundary items), in accordance with predictions from event segmentation theory (Radvansky & Zacks, 2017; Zacks, 2020). A second aim of the encoding task was to enhance temporal order memory accuracy. This would allow us to test whether the findings from Experiment 1 indicated generalizable insights in temporal memory processing or that they were limited by poor memory performance.

## Experiment 2: Methods and procedures

### Participants

Twenty-five participants (19 females, mean age = 21.2 years, SD = *2.22* years) were recruited via online social media and a student database at the Faculty of Psychology and Neuroscience. In addition to exclusion criteria of Experiment 1, participants were also excluded when they could or would not download, install, or run the experiment on a computer at home (due to government imposed social distancing regulations, see below). The study was approved by the local ethics review board of the Faculty of Psychology and Neuroscience. All participants gave online informed consent prior to the start of the experiment and received course credit or financial compensation for their participation.

### Materials and procedures

Experiment 2 used the same materials as in Experiment 1. At the time of Experiment 2 (February–April 2021), the Dutch government imposed social distancing and lockdown regulations that largely prohibited students from visiting university laboratories. Therefore, to conduct this experiment, we asked participants to complete it at home by installing PsychoPy (Bridges et al., 2020) and downloading the experiment code to a computer that was available to them at home. The experiment procedures were the same as those for Experiment 1, with the following exceptions.

During the encoding phase, participants were instructed for each item to rate whether the ISI *before* the item "feels appropriate" to that item. For example, a short ISI might feel more appropriate to images of an anthill or a race car, while a long ISI might feel more appropriate to an image of a mountain or a snail. Participants had to give their rating during the 2,250 ms that an item was presented on the screen by pressing "1" for "feels appropriate" or "2" for "does not feel appropriate" on their keyboard. After each list, participants completed the temporal order memory and temporal source memory tasks in a similar way as in Experiment 1, but with the exception that responses were self-paced (thereby avoiding speed–accuracy trade-offs altogether). In each memory task, the visual items remained on the screen until button press.

### Analysis

To investigate response times in the encoding task as a function of temporal boundaries, we analyzed boundary (first item of a timing context) and nonboundary serial position (remaining items of a timing context), collapsed across ISIs. Temporal order and temporal source memory responses were analyzed in the same way as for Experiment 1.

### Results

#### Encoding task

**Fig. 4:**
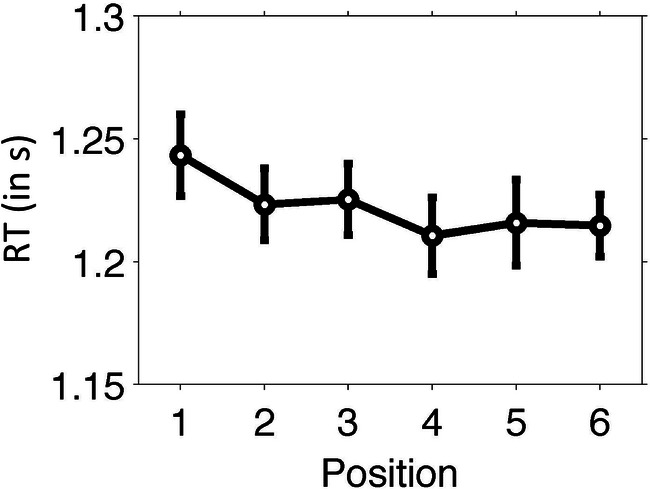
Encoding results of Experiment 2. RTs during encoding increased with longer ISI durations. Error bars indicate 95% confidence interval (Masson & Loftus, 2003)

Figure 4 depicts the encoding RTs per serial position (collapsed across ISIs). To test the effect of boundary position within a timing context, we compared RT of the first item (temporal boundary item) to that of the remaining (nonboundary) items. Boundary RT (*M* = 1.24, *SD* = 0.22) was significantly higher, *t*(24) = 2.63, *p* = .015, Cohen’s *d* = 0.53, than nonboundary RTs (pooled across non-boundary positions, *M* = 1.22, *SD* = 0.23), indicating that a change in timing context acted as an event boundary during encoding.

To assess the pattern of differences between boundary and non-boundary items, we conducted post hoc comparisons (paired-sample *t* tests, not corrected for multiple comparisons). Boundary RT was not significantly higher for the first two boundary items, Position 2: *t*(24) = 1.98, *p* = .059; Position 3: *t*(24) = 1.46, *p* = .16, but was significantly higher for subsequent nonboundary items, Position 4: *t*(24) = 2.49, *p* = .02; Position 5: *t*(24) = 2.12, *p* = .045; Position 6: *t*(24) = 2.60, *p* = .016. A linear polynomial contrast that captured the gradual decrease of RTs over serial position was significant (*p* = .009). These findings appear consistent with a smearing of the effect of temporal boundaries on encoding responses from the boundary to nearby nonboundary items.

#### Temporal order memory

**Fig. 5:**
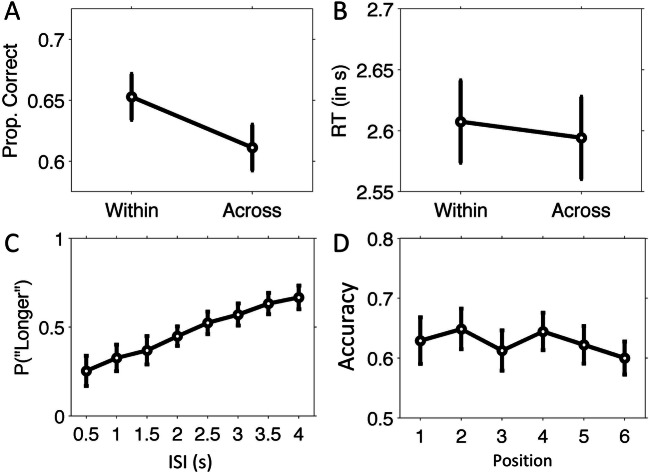
Temporal memory results of Experiment 1. Temporal order memory accuracy (proportion correct; **a**) and RTs (in seconds; **b**). Proportion “longer” responses (**c**) and accuracy of the temporal source memory task (**d**). For clarification, see caption of Fig. 2. Error bars indicate 95% confidence intervals (Masson & Loftus, 2003)

Figure 5a shows the mean proportions of the correct answers of the within-context and across-context conditions of the temporal order task. Within-context accuracy (*M* = .65, *SD* = .08) was significantly higher than across-context accuracy (*M* = .61, *SD* = .08), *t*(24) = 2.28, *p* = .032, Cohen’s *d* = .46. None of the participants had an overall average performance below 0.5. Thus, the active encoding task resulted in higher overall temporal order memory performance while also eliciting enhanced performance of the within-context relative to the across-context condition. Response times did not significantly differ between the two conditions, *F*(1, 24) = .16, *p* = .69 (see Fig. 5b).

We also compared accuracy for Within-context temporal order judgments of short ISIs to that of long ISIs (excluding ISIs of 2.0 and 2.5). Results showed that temporal order accuracy for short ISIs (*M* = .66, *SD* = .12) was again lower than for long ISIs (*M* = .67, *SD* = .11), but this difference was not significant (*p* = .31, one-tailed).

#### Temporal source memory

Figure 5c depicts the proportion “longer” responses that participants made on average for items from each of the eight ISIs in Experiment 2. Results were similar to those of Experiment 1, with the lowest proportion of “longer” responses occurring during the shortest interval of 0.5 s, and progressively increased for longer intervals. A one-way repeated-measures ANOVA showed that this effect was significant (Greenhouse–Geisser corrected), *F*(2.8, 65.5) = 30.01, *p* < .001, ${\eta}_p^2$ = .57, as was its linear contrast (*p* < .001). Proportions were significantly below 0.5 (one-sample *t* tests) for intervals of 0.5, 1, and 1.5 s, and significantly higher for intervals 3.0, 3.5, and 4 s (see Table 2). We also analyzed whether the association between items and ISIs differed across item position, and found no significant effect, *F*(5, 115) = 1.14, *p* = .34; see Fig. 5d).

**Table 2:** Temporal source memory results Experiment 2 (for explanatory notes, see Table 1**)**

ISIs	Mean	*SE*	*t*	*p*	Cohen's *d*
0.5	0.25	0.04	−6.01	<0.001	−1.23
1.0	0.33	0.04	−4.83	<0.001	−0.99
1.5	0.37	0.04	−3.41	0.002	−0.70
2.0	0.45	0.03	−1.91	0.068	−0.39
2.5	0.52	0.03	0.79	0.439	0.16
3.0	0.57	0.03	2.37	0.027	0.48
3.5	0.63	0.03	4.56	<0.001	0.93
4.0	0.67	0.03	5.19	<0.001	1.06

**Fig. 6:**
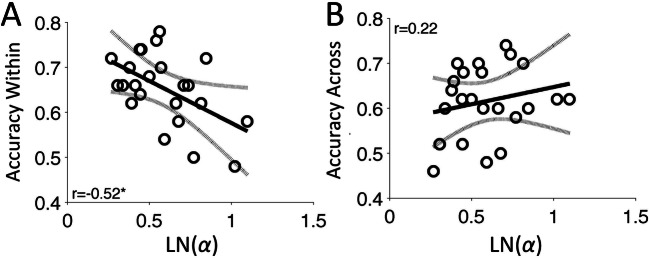
Temporal memory task correlations of Experiment 2. For clarification, see caption of Fig. 3. Error bars indicate 95% confidence intervals. *p < .05

We then repeated the analysis of the relation between the psychometric curves and temporal order memory accuracy, following the same procedure as described for Experiment 1. For the log-normalized shape parameter, ln*(α)*, the average (*SD*) was 0.61 (0.25), with a range of 0.27–1.18. The shape parameter was again significantly negatively correlated with Within-context temporal order judgments (*r* = −.52, *p* = .01; see Fig. 6a), but not with across-context order judgments (*r* = .22, *p* = .32; see Fig. 6b), and these correlations were significantly different (*z* = −2.85, *p* = .002, two-tailed). These results replicate those of Experiment 1, and suggest that the relation between temporal underestimation and temporal order judgments due to event segmentation did not depend on how the stimuli were encoded.

For the log-normalized slope parameter, ln(*β*), the average (*SD*) was −0.32 (0.87), with a range of −3.10–0.27. The slope parameter correlated significantly with within-context judgments (*r* = .54, *p* = .007), but not with across-context judgments (*r* = .13, *p* = .57). The two correlation coefficients did not significantly differ (*z* = 1.72, *p* = .08, two-tailed).

### Discussion

The inclusion of an encoding task in Experiment 2 replicated as well as extended the findings of Experiment 1. The encoding task revealed elongated response times for boundary items, but this effect appeared to be smeared to nearby nonboundary items as well. These findings are in line with the suggestion that temporal boundaries are weaker or smeared across time, compared to visual boundaries, as changes in timing context may take some time to be detected or processed. Notably, these findings differ from temporal boundaries elicited by symbolic cues (Ezzyat & Davachi, 2011; Magliano et al., 2001; Speer & Zacks, 2005), suggesting different encoding mechanisms for temporal contextual changes that are implied or when actually perceived. These findings indicate that, on the one hand, contextual changes elicited longer response times for boundary items, in line with event segmentation theory (Radvansky & Zacks, 2017; Zacks, 2020), while on the other hand changes in temporal contexts are processed differently than changes in visual contexts.

Further, the encoding task resulted in better overall temporal order memory performance, as well as enhanced temporal order memory for within-context items, compared with items crossing a temporal boundary. This finding replicates temporal order memory effects reported in event segmentation studies using implied temporal contextual changes (Ezzyat & Davachi, 2011) as well as nontemporal contexts (DuBrow & Davachi, 2013, 2014; Heusser et al., 2018), and suggests that temporal boundaries affect mnemonic processing similarly as nontemporal boundaries.

Finally, we replicated the correlation between temporal underestimation and context-based temporal order judgments of Experiment 1. Moreover, we found that the positive correlation between the slope parameter and within-context order judgments, which was close to significant in Experiment 1, reached significance in Experiment 2. Our replication substantiates the findings of Experiment 1 and indicates that individual differences in temporal processing play a role in event segmentation based on timing contexts.

## General discussion

We argue that both experiments reveal evidence of event segmentation based on temporal contexts. The longer response times for across-context temporal order judgments in Experiment 1 suggest that participants had more difficulty with temporal order judgments for items that were encoded in different temporal contexts compared with items encoded in the same context. This response time pattern appears reminiscent of studies that showed longer response times in memory recognition when participants had to search through larger item sets in memory (Nosofsky et al., 2014; Wolfe, 2012). When projected to our findings, the longer RTs for across-context judgments could indicate an expanded memory search across different memory sets, if one assumes that items were segmented into separate events based on the changes in timing context. The effect cannot be explained by differences in perceptual set size, as the actual list lengths and distance between items (i.e., number of intervening items) for order judgments were the same for the two temporal order conditions. Thus, the difference in RTs supports the role of timing context in event segmentation from a memory search perspective. Arguably, the absence of a contextual temporal order effect of accuracy in Experiment 1 could indicate that, while memory for the items was weak, temporal boundaries nonetheless segmented the visual items according to temporal contexts in memory. The encoding and temporal order memory results of Experiment 2 underscore this notion.

Further, our findings fit with the suggestion that individual differences in mental time keeping affect temporal order memory and, by proxy, event segmentation. Internal clock models suggest that a stronger temporal underestimation bias results from a relatively slower clock speed that accumulates clock ticks at a slower rate. Participants with a stronger underestimation bias tend to experience or judge a time interval to last shorter than it actually does. Consequently, slower mental clocks would make it more difficult to discriminate between time intervals that are relatively close in duration, compared with faster mental clocks, and would ultimately hamper the detection or processing of temporal boundaries. As event boundaries play a pivotal role in event segmentation and clustering of temporally organized items within a context (DuBrow & Davachi, 2014; Radvansky & Zacks, 2017; Zacks, 2020), temporal underestimation could thus impair temporal associative binding within contexts.

Temporal underestimation could also affect mnemonic processing more directly. Temporal features of events that were perceived as occurring closely in time could be represented more similarly in memory, which would make it more difficult to temporally differentiate those events in memory. This reasoning builds on findings of increased memory errors or confusion when (nontemporal) perceptual or mnemonic features of distinct events are perceived as more similar (Criss, 2006; Oberauer & Lange, 2008). Some evidence in support of temporal similarity comes from functional magnetic resonance imaging (fMRI) studies showing that brain activity patterns for distinct items or events becomes more similar when they are perceived or remembered as having occurred more closely in time, compared with when they are perceived or remembered as more distant in time (Ezzyat & Davachi, 2014; Lositsky et al., 2016). A stronger temporal underestimation bias would result in more similar temporal features and thus lead to impaired within-context temporal order judgments. However, this account does not explain the poor temporal order performance for across-context judgments, in which temporal similarity is minimized.

The psychometric function was obtained from subjective duration judgments based on temporal source memory. To our knowledge, we are the first to use this method to investigate temporal memory bias of subjective durations. However, as we did not measure subjective judgments of perceived durations (that is, judgments about physically varying durations), we could not ascertain whether mnemonic duration bias differed from perceptual duration bias. Previous studies have shown that perceptual durations may compress in episodic memory (Furman et al., 2007; Jeunehomme et al., 2018), which leaves open the possibility that our observed correlation may be driven by the common mnemonic source of the judgments, which may be independent of the rate of a mental clock. Nevertheless, our findings do show that a change in timing context can affect contextual processing of a series of events in memory.

Finally, it has previously been shown that rhythmic presentation of items enhances subsequent recognition over nonrhythmic presentations (Jones & Ward, 2019). This effect may be related to enhanced stimulus detection and attentional encoding when items are presented rhythmically (Bolger et al., 2014; Rohenkohl et al., 2011; Rohenkohl et al., 2012), which in turn owes to the predictability that is inherent to rhythmic stimulus presentation and facilitates temporal attentional deployment (Lamy, 2005; Martens & Johnson, 2005; Nobre & van Ede, 2018; van Ede et al., 2017; Vangkilde et al., 2012). This was further demonstrated by Thavabalasingam et al. (2016), who showed enhanced memory when item series with varying interstimulus intervals between the items were shown multiple times with the same temporal structure, indicating that the inherently arrhythmic temporal structure became predictable over repetitions. In turn, other studies showed that temporal expectations that were retrieved from memory could affect recognition performance of subsequently presented items (Cravo et al., 2017; van de Ven et al., 2017). Our findings contribute to these lines of research by showing that changes in timing contexts may also serve to segment and bind items within a context.

In conclusion, we showed that timing context can support event segmentation, and that the subjective duration bias from temporal source memory was inversely correlated to the accuracy of within-list temporal order judgments. Our results have ramifications for understanding how humans segment temporally organized experiences in memory.
